# Developing a Clear and New Definition of Non-specific Abdominal Pain for the Identification of Rare Diseases

**DOI:** 10.7759/cureus.96263

**Published:** 2025-11-06

**Authors:** Masaki Tago, Naoko E Katsuki, Risa Hirata, Susumu Tazuma

**Affiliations:** 1 General Medicine, Saga University Hospital, Saga, JPN; 2 Internal Medicine and Healthcare Management and Administration, JR Hiroshima Hospital, Hiroshima, JPN

**Keywords:** acute abdominal pain, definition, non-specific abdominal pain, rare disease, undiagnosed abdominal pain

## Abstract

Non-specific abdominal pain (NSAP) represents a diagnostic challenge that may include various rare diseases requiring early identification. If NSAP can be clearly defined, it may enable the early diagnosis of such rare diseases. This study aimed to develop a definition of NSAP based on the characteristics of diagnoses related to abdominal pain and on the backgrounds of undiagnosed patients in our previous study, as well as to provide recommendations. Data concerning patients with undiagnosed abdominal pain were analyzed to determine appropriate diagnostic criteria and timeframes for the NSAP definition. Based on this analysis, we propose the following definition of NSAP: "Abdominal pain that remains undiagnosed for ≥7 days after onset, despite undergoing abdominal computed tomography or ultrasonography, blood tests, and a general qualitative urine test. The abdominal pain may be persistent or intermittent and recur for ≥7 days. The duration and interval of intermittent pain are not considered." This proposed definition of NSAP may aid in identifying patients with rare diseases that cause abdominal pain.

## Introduction

Many patients present to outpatient clinics with abdominal pain as their chief complaint [1]. In emergency departments, non-specific abdominal pain (NSAP) and urinary stones are the most common causes of abdominal pain (each accounting for approximately 31% of cases) [2]. To date, the definition of NSAP remains unclear, with studies using various definitions, such as abdominal pain that cannot be classified as a specific disease [2], undifferentiated abdominal pain [3,4], and abdominal pain during hospitalization that does not require emergency surgical intervention because the direct cause cannot be identified [5]. With the recent proliferation and advancement of diagnostic equipment, diagnostic techniques have improved, and the proportion of patients classified with NSAP has decreased [3]. Rare diseases, such as acute hepatic porphyria (AHP), hereditary angioedema, Fabry disease, inflammatory bowel disease, lead poisoning, desmoid fibromatosis, and mastocytosis, may be included in NSAP. These rare diseases, including complex diseases with multiple symptoms, metabolic diseases, and genetic diseases, involve small numbers of patients and are not generally known to clinicians. For example, AHP is characterized by abdominal pain as the most common symptom, which may take over a decade to diagnose [6]. Therefore, abdominal pain is often initially considered to be NSAP or misdiagnosed as another disease until an accurate diagnosis is made. Some rare diseases causing abdominal pain were previously considered to have a poor prognosis; however, new and effective treatments, such as those for AHP, have recently been developed. Rare diseases, such as AHP, have a high probability of remaining undiagnosed and persisting as undiagnosed abdominal pain (UDAP). These diseases are likely to be included in our proposed NSAP definition. If NSAP can be clearly defined, early diagnosis of rare diseases may be facilitated.

In light of UDAP, we conducted a survey on the characteristics of abdominal pain diagnoses and the background of undiagnosed patients in previous studies [7]. This study aimed to develop a definition of NSAP based on these data and to make recommendations for diagnosing rare diseases included in NSAP.

## Technical report

Material and methods

This sub-analysis used data from a previous retrospective observational study that targeted patients who had undergone imaging examinations (abdominal computed tomography (CT), ultrasonography, upper gastrointestinal endoscopy, or lower gastrointestinal endoscopy) between April 1, 2019, and March 31, 2022. These patients had visited the general medicine departments of six acute general hospitals with emergency departments in Japan (median number of beds, 915; interquartile range, 549-1087) [7]. We included patients with abdominal pain who met both examination and department criteria within the target period. We excluded patients who declined to participate in this study. The patients’ medical records were retrospectively reviewed for detailed information regarding abdominal pain, age, sex, time from the onset of abdominal pain to diagnosis, and the presence or absence of examinations such as blood tests, urine tests, and electrocardiograms. Two doctors from each facility individually reviewed the medical records and determined the final diagnosis. Patients without a diagnosis were classified into a UDAP group. Six specialists in general medicine conducted a focus group discussion to reach a consensus regarding the time taken for a patient to be classified as having UDAP, the type of imaging examination included in the inclusion criteria, and the implementation rate of blood or urine tests. Based on the results of that discussion, the definition of NSAP was determined. The group of experts made clinically reasonable decisions, such as adopting examination items intended for use in a wide range of clinical settings, based on a proposal to adopt items with implementation rates of ≥80% and medians for continuous variables. Through this collaborative discussion process, the specialists established agreed-upon standards and finalized the results based on their collective expertise and on consensus. Descriptive statistics were performed using statistical analysis software R (version 4.2.2; R Foundation for Statistical Computing, Vienna, Austria).

Results

Our previous study included 1,915 patients, of whom 317 (16.6%) were classified into the UDAP group [7]. The implementation rates for imaging examinations in the UDAP group are shown in Table 1. The median age in the UDAP group was 55 years, and 42.3% of the patients were male. Abdominal ultrasonography, abdominal plain radiography, abdominal CT, abdominal magnetic resonance imaging, upper gastrointestinal endoscopy, lower gastrointestinal endoscopy, and transvaginal ultrasonography were performed in 30.6%, 35.3%, 92.7%, 4.1%, 15.8%, 13.6%, and 2.2% of the patients, respectively. Electrocardiograms, urine tests, and blood tests were performed in 44.2%, 62.5%, and 95.6% of the patients, respectively. The specific results and implementation rates of the complete blood tests are shown in Table 2. Among the complete blood test items, white blood cell (WBC) count, leukocyte classification, red blood cell (RBC) count, hemoglobin (Hb), hematocrit (Ht), mean corpuscular volume (MCV), mean corpuscular hemoglobin (MCH), mean corpuscular hemoglobin concentration (MCHC), platelet count (PLT), aspartate aminotransferase (AST), alanine aminotransferase (ALT), gamma-glutamyl transferase (γGTP), alkaline phosphatase (ALP), total bilirubin (T-Bil), lactate dehydrogenase (LDH), total protein (TP), albumin (Alb), blood urea nitrogen (BUN), creatinine (Cr), estimated glomerular filtration rate (eGFR), amylase (AMY), sodium (Na), potassium (K), chlorine (Cl), and C-reactive protein (CRP) levels were evaluated in >80% of the patients.

**Table 1:** Implementation rate of examinations in the UDAP group UDAP, undiagnosed abdominal pain Source: [7]

Performed examination	Implementation rate in the UDAP group
Abdominal ultrasonography	30.6%
Abdominal computerized tomography	92.7%
Abdominal magnetic resonance imaging	4.1%
Abdominal plain radiography	35.3%
Upper gastrointestinal endoscopy	15.8%
Lower gastrointestinal endoscopy	13.6%
Transvaginal ultrasonography	2.2%
Electrocardiography	44.2%
Urine test	62.5%
Blood test	95.6%

**Table 2:** Implementation rate of the performed blood tests Source: [7]

Blood tests performed	Implementation rate in the UDAP group
Aspartate aminotransferase	84.2%
Alanine aminotransferase	95.0%
Gamma-glutamyl transferase	94.6%
Alkaline phosphatase	89.3%
Total bilirubin	91.5%
Lactate dehydrogenase	94.0%
Total protein	86.4%
Albumin	89.0%
Albumin-globulin ratio	34.7%
Choline esterase	43.8%
Hepatitis B surface antigen	21.5%
Hepatitis B surface antibody	10.7%
Hepatitis C virus antibody	22.1%
Triglyceride	43.8%
High-density lipoprotein cholesterol	37.2%
Low-density lipoprotein cholesterol	37.9%
Glucose	76.7%
Hemoglobin A1c	38.5%
Urinary acid	56.8%
C-reactive protein	93.4%
Blood urea nitrogen	93.7%
Creatinine	94.0%
Estimated glomerular filtration rate	93.1%
Amylase	80.1%
White blood count	95.0%
Leukocyte classification	92.7%
Red blood cell	95.0%
Hemoglobin	95.0%
Hematocrit	95.0%
Mean corpuscular volume	95.0%
Mean corpuscular hemoglobin	95.0%
Mean corpuscular hemoglobin concentration	95.0%
Platelet	95.0%
Sodium, potassium, chlorine	94.3%
Calcium	58.0%
Magnesium	5.4%
Alpha-fetoprotein	0.9%
Carcinoembryonic antigen	11.0%
Prostate-specific antigen	0.9%
Carbohydrate antigen 125	3.8%
Carbohydrate antigen 15-3	0.6%
Carbohydrate antigen 19-9	9.8%
Blood gas test	43.5%

The time from onset to definitive diagnosis was calculated as the time from onset to the date of the final visit. The median time to classification as UDAP was 6.4 days (interquartile range, 1.7-75).

**Figure 1:**
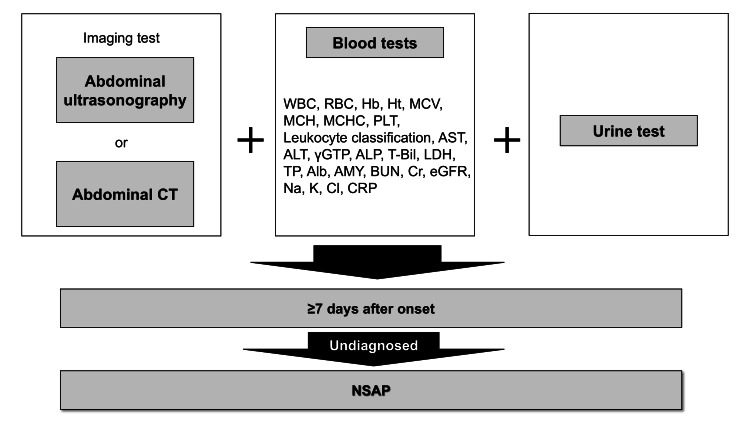
Process to determine NSAP Alb, albumin; ALP, alkaline phosphatase; ALT, alanine aminotransferase; AMY, amylase; AST, aspartate aminotransferase; BUN, blood urea nitrogen; Cl, chlorine; Cr, creatinine; CRP, C-reactive protein; CT, computed tomography; eGFR, estimated glomerular filtration rate; Hb, hemoglobin; Ht, hematocrit; K, potassium; LDH, lactate dehydrogenase; MCH, mean corpuscular hemoglobin; MCHC, mean corpuscular hemoglobin concentration; MCV, mean corpuscular volume; Na, sodium; NSAP, non-specific abdominal pain; PLT, platelet count; RBC, red blood cell; T-Bil, total bilirubin; TP, total protein; WBC, white blood count; γGTP, gamma glutamyl transferase Note: This figure shows an algorithm for when patients present with abdominal pain. NSAP is defined as abdominal pain in patients who have undergone abdominal US or abdominal CT, who have also undergone blood tests and urine tests, and who remain undiagnosed for ≥7 days after onset.

Based on these results, we propose the following definition for NSAP: “Abdominal pain that remains undiagnosed for ≥7 days after onset, despite the implementation of abdominal computed tomography or abdominal ultrasonography, the following complete blood tests (including WBC, leukocyte classification, RBC count, Hb, Ht, mean corpuscular volume (MCV), mean corpuscular hemoglobin (MCH), mean corpuscular hemoglobin concentration (MCHC), platelet (PLT), aspartate aminotransferase (AST), alanine aminotransferase (ALT), gamma-glutamyl transferase (γGTP), alkaline phosphatase (ALP), total bilirubin (T-Bil), lactate dehydrogenase (LDH), total protein (TP), albumin (Alb), blood urea nitrogen (BUN), creatinine (Cr), estimated glomerular filtration rate (eGFR), amylase (AMY), sodium (Na), potassium (K), chlorine (Cl), and C-reactive protein (CRP) levels), and a general qualitative urine test. The abdominal pain may be persistent or intermittent and recur for ≥7 days after the initial onset. The duration and interval of intermittent pain are not considered.” (Figure 1).

## Discussion

We determined a new definition of NSAP derived from examinations using data from previous research. This definition is based on real clinical data, can be used in any clinical setting, and enables clinical screening for patients with UDAP. In this study, UDAP was not compared with NSAP. Our definition of NSAP excluded mild abdominal pain that occurred only once and did not require a diagnosis. We aimed to focus on abdominal pain that requires a diagnosis and unspecified UDAP that has repetitive symptoms or persists for a prolonged period. Therefore, it is important to distinguish between NSAP and abdominal pain, which is included in UDAP and does not require a diagnosis. Our definition, which includes the use of blood and urine tests and a time frame that can be objectively evaluated, seems reasonable based on data from our clinical practice. Imaging tests frequently performed to diagnose patients with abdominal pain include abdominal plain radiography, abdominal ultrasonography, and abdominal computed tomography (CT) [8,9]. In Japan, most hospitals use CT technology, which is relatively easy to apply. In this study, which was conducted in large-scale hospitals, the implementation rate of CT for UDAP was >90%. However, as the implementation rate of abdominal ultrasonography was low, and considering that some hospitals cannot perform CT outside business hours or in clinics, we decided to include ultrasound examinations, which are even easier to perform and less invasive than CT. The sensitivity and specificity of abdominal plain radiography, abdominal ultrasound, and CT for detecting organic lesions are reported to be 28% and 91%; 61.8% and 98.4%; and 87.8% and 92.2%, respectively [8]. Abdominal ultrasonography has the second-highest sensitivity and specificity after CT, and by including abdominal ultrasonography in the definition, the application range of this definition can be significantly expanded. Therefore, it is reasonable to include abdominal ultrasonography in the definition of NSAP.

We included blood tests performed in >80% of patients with UDAP and a simple qualitative urine test according to the definition of NSAP. In blood tests, WBC counts and CRP levels are known to be useful for diagnosing acute appendicitis [10], and a complete blood count is widely used for evaluating patients with abdominal pain [11]. In patients with right upper abdominal pain, those with liver function abnormalities, and those with upper abdominal pain, AMY measurement can contribute to the diagnosis [12]. Moreover, a model predicting the severity of acute abdominal pain has included WBC count, Ht, glucose, BUN, Cr, Alb, AST, T-Bil, conjugated bilirubin, and AMY levels [13]. Therefore, blood tests contribute significantly to the diagnosis of abdominal pain. Additionally, in the treatment of patients with non-traumatic abdominal pain, many medical professionals consider qualitative urine tests useful [14]. Qualitative urine tests are suitable for inclusion in the definition of NSAP because they contribute to the diagnosis of hematuria [15], ketone bodies [16], and bilirubin [17], and can be performed easily with minimal invasiveness.

Among the patients classified as NSAP and without a diagnosis during hospitalization in the emergency department, 10 of 249 patients who were discharged with NSAP (4%) received a diagnosis within 3 days after discharge, and 9 (3.6%) patients reportedly received a diagnosis within 3 months [18]. If the time from onset is too short, there may be insufficient information for diagnosis. Considering the possibility that NSAP includes diseases that should be diagnosed promptly, this definition may be ineffective if the period until NSAP diagnosis is too long. The median time from onset to confirmation of UDAP in patients who presented with abdominal pain was 6.4 days; thus, being undiagnosed for ≥7 days was included in the definition of NSAP.

Study limitations

A new definition of NSAP was created using data from a retrospective observational study. However, because the implementation of imaging examinations is a criterion for inclusion, it is possible that not all patients who presented with abdominal pain were included, and there could have been selection bias. This study’s data were obtained from six large acute care Japanese hospitals, which may have led to bias in the patient population. It is possible that this definition cannot be readily generalized and that there is potential misclassification bias.

## Conclusions

The new definition of NSAP that we developed, based on frequently performed examinations in patients with UDAP, is grounded in data from our clinical practice and empirical reasoning from the authors. The proposed definition of NSAP may aid in identifying patients with rare diseases that cause abdominal pain such as AHP and hereditary angioedema. Future research on such patients could lead to the establishment of diagnostic methods for rare diseases that present with abdominal pain. However, our proposed definition of NSAP remains to be verified, and we cannot definitively state that it necessarily includes rare diseases. Therefore, future prospective research is needed for further verify our findings.
